# Complete mitochondrial genome of green shrimp, *Chlorotocus crassicornis* (Crustacea: Decapoda: Pandalidae) in Korean water

**DOI:** 10.1080/23802359.2019.1624633

**Published:** 2019-07-11

**Authors:** GyungRyul Kim, Md. Jobaidul Alam, Hyun-Woo Kim, Sapto Andriyono

**Affiliations:** a Fisheries Resources Research Division, National Institute of Fisheries Science (NIFS), Busan, South Korea;; b Interdisciplinary program of Biomedical Engineering, Pukyong National University, Busan, South Korea;; c Department of Marine Biology, Pukyong National University, Busan, Korea;; d Department of Marine, Fisheries and Marine Faculty, Universitas Airlangga, C Campus Jl, Mulyorejo Surabaya East Java, Indonesia

**Keywords:** *Chlorotocus crassicornis*, Pandalidae, crustacean, mitochondrial genome

## Abstract

The complete mitochondrial genome of green shrimp, *Chlorotocus crassicornis* (Costa, 1871) was generated by the combination of next-generation sequencing platform and long PCR technique. The mitochondrial genome of *C. crassicornis* was 16,500 bp, in which 13 protein-coding genes, two ribosomal RNAs, 22 transfer RNAs, and a putative control region was encoded. Based on the 13 protein-coding genes region, the phylogenetic tree was clearly demonstrated that *C. crassicornis* is closest to *Pandalopsis japonica* and *Pandalus borealis* with 77% identity. This mitogenome information will be helpful for the further studies of deep-sea fisheries resources management strategies in Korea including *C. crassicornis* species.

The green shrimp, *Chlorotocus crassicornis* (Costa, 1871) inhabits in the deep water (200–400 m) (Cartes 1993), which was originally identified in the Mediterranean and the Atlantic Ocean, Almeria Canyon (Würtz 2012), its widely distributed from the Mediterranean Sea to the Indo-West Pacific (Li 2006; De Grave and Fransen 2011). This species plays an important ecological role as a benthic feeder (Cartes 1993). We first determined the complete mitochondrial genome of green shrimp belonging to *C. crassicornis* (GenBank accession number NC 035828).

The *C. crassicornis* was collected from the East China Sea (123.5°E and 34.5°N), and the specimen has been deposited at the National Institute of Fisheries Science (NIFS), Korea. The complete mitochondrial genome was obtained by assembling two large PCR fragments using long-PCR technique. Then, the PCR products were pooled together and fragmented into 350 bp by Covaris^®^ M220 (Covaris Inc, Woburn, MA, USA). In order to run on the Illumina Miseq platform (Illumina, San Diego, USA), a library was constructed using TruSeq^®^ RNA library preparation kit V2 (Illumina, San Diego, USA) according to the manufacturer’s instructions. After PCR enrichment step, the products were loaded on 2% agarose gel, the products from 500bp to 700 bp were cut and purified with MiniElute PCR purification kit (Qiagen, Hilden, Germany). Finally, the library was sequenced by the MiSeq sequencer using 300 × 2 paired-end reads. The raw reads were trimmed and low quality reads (QV < 20) were decollated by using CLC Genomic Workbench v.8.0 (CLC Bio, Cambridge, MA) and followed Mothur software (v.1.36.1) to analyze of mismatch between paired (Schloss et al. 2009). The paired sequences were assembled using the Geneious R11.0.2 (Kearse et al. 2012).

The complete mitochondrial genome of *C. crassicornis* was 16,500 bp in length, which was similar to her relative, *Pandalopsis japonica* and *Pandalus borealis* (Viker et al. 2006). The mitogenome contained 22 tRNAs, two ribosomal RNAs (12S and 16S), 13 protein-coding genes, and two non-coding regions; and a putative control region (*D-Loop*). Among 37 genes, 23 genes (nine protein-coding genes, and 14 tRNAs) were encoded on the H-strand, while the remaining 14 genes (two rRNAs, ND1, ND4, ND4L, ND5, and eight tRNA genes) were located on the L-strand. All tRNAs were predicted to be a typical three folded clover-leaf structure, except tRNA*^Ser^* (Laslett and Canbäck 2008). The start codon was not determined at the COX1 region, similar to other crustacean *Pleoticus muellerii* (Kim et al. 2018), the typical start codon (ATG) was initiated at six PCGs (COX2, ATP6, COX3, ND4, ND4L, and *Cyt b*). The incomplete stop codon (TA––/T––) were identified in five genes including COX1, COX2, ND5, ND4, and *Cytb*.

A minimum-evolution (ME) phylogenetic tree (Kumar et al. 2016) of 14 species within the suborder Pleocyemata was constructed based on 13 PCGs. The phylogenetic tree was clearly demonstrated that *C. crassicornis* closest to *P. japonica* (Yoon et al. 2019) and *P. borealis* with 77% identity (Figure 1). This mitogenome information would contribute to phylogenetic studies of *C. crassicornis* and further deep-sea fisheries resources management strategies in Korea.

**Figure 1.**
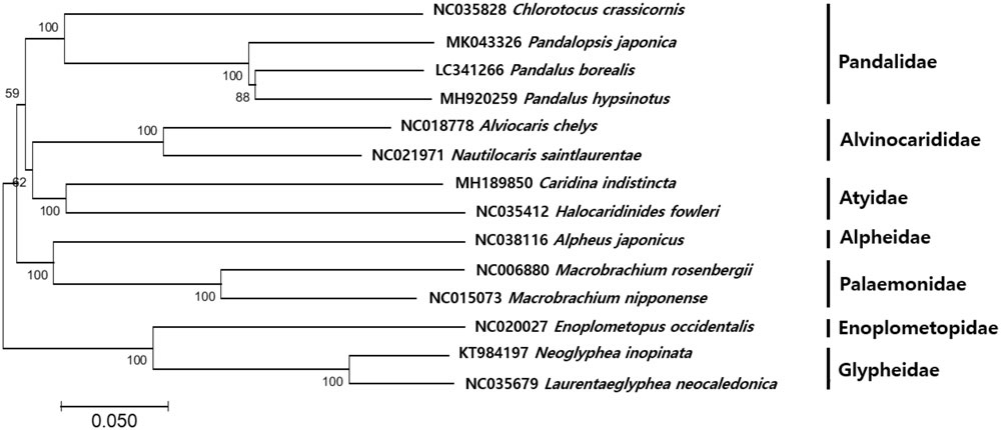
Phylogenetic relationship of *Chlorotocus crassicornis* within Pandalidae.

Phylogenetic analysis of *C. crassicornis* was constructed with the mitogenome sequences using MEGA7 software with Minimum Evolution (ME) algorithm with 1000 bootstrap replications. GenBank Accession numbers were shown followed by each scientific name.
